# Neuroepithelial cells from metformin‐treated patients exhibit a preconditioned response to metformin in vitro

**DOI:** 10.1002/alz70856_105315

**Published:** 2026-01-08

**Authors:** Francisco‐Alexei Ramirez‐Galvan, Diana‐Angelica Jimenez‐Galaviz, Juan‐Ramon Padilla‐Mendoza, Rafael Jijon‐Lorenzo, Maria‐del‐Carmen Silva‐Lucero, Maria‐del‐Carmen Cardenas‐Aguayo

**Affiliations:** ^1^ UNAM, School of Medicine, Department of Physiology, CDMX, DF, Mexico; ^2^ División de Químico Biológicas, Universidad Tecnológica de Tecámac, Mexico, EM, Mexico

## Abstract

**Background:**

Type 2 Diabetes (T2D) is highly prevalent in Latin America, particularly in Mexico, with 15.6% of the population affected (ENSANUT 2020, Montoya 2023). T2D increases the risk of complications: hypertension and dementia. Mild Cognitive Impairment (MCI) precedes Alzheimer's dementia, the most common type.

Metformin, the primary treatment for T2D, enhances autophagy, a cellular process for recycling primary components. Autophagy impairment is linked to dementia (Nixon 2024).

This study utilizes human olfactory neuroepithelial precursor cells (hONE‐NPCs) as a model for neurodegeneration. Olfactory dysfunction is an early indicator of dementia, suggesting that alterations in hONE‐NPCs may reflect early stages of the disease.

**Method:**

This study included four groups: healthy controls, individuals with cognitive impairment, those with T2D, and with T2D and cognitive impairment (T2D‐MCI). hONE NPCs were isolated from each participant and characterized using Western Blotting and immunocytofluorescence. Blood samples were collected for HbA1c and protein samples for autophagy markers.

Participants underwent a MoCA Test by a certified applicator (MXRAMFR710802563‐01), a medical interview to assess metformin treatment history, and an olfactory test. hONE‐NPCs were treated with or without metformin for 24 hours. Protein samples were collected for Western Blotting analysis in three groups in vitro: Diabetes+MCI+Metformin, Diabetes‐MCI‐Metformin, and Control+Metformin.

**Result:**

Culture cells hONE‐NPCs from Diabetes+MCI and Pre‐Diabetic+MCI from patients that had been treated with metformin and their cells were treated with metformin show an impairment in the autophagy flux since p62 was elevated and LC3‐II was downregulated. Conversely, culture cells from control individuals who had never been treated with metformin responded to metformin in vitro treatment with an increase in autophagy flux, showing p62 reduction and an increase in LC3‐II.

**Conclusion:**

Our results suggest a preconditioning effect of hONE‐NPCs from patients receiving metformin treatment on their response to this drug in vitro. Cells from patients who were prescribed metformin show an impairment in the autophagy flux; however, culture hONE.NPCs cells from control individuals who had never been treated with metformin responded to metformin in vitro treatment with an increase in autophagy flux. This finding may have clinical implications in the long‐term effects of metformin treatment in peripheral cells as a systemic effect.

